# Benefits of macroscopic on-site evaluation in endoscopic ultrasound-guided tissue acquisition for comprehensive genomic profiling

**DOI:** 10.1055/a-2593-4172

**Published:** 2025-05-16

**Authors:** Junya Sato, Hirotoshi Ishiwatari, Kazuma Ishikawa, Hiroki Sakamoto, Takuya Doi, Masahiro Yamamura, Kazunori Takada, Yoichi Yamamoto, Masao Yoshida, Sayo Ito, Noboru Kawata, Kenichiro Imai, Kinichi Hotta, Hiroyuki Ono

**Affiliations:** 1 38471 Division of Endoscopy, Shizuoka Cancer Center, Sunto-gun, Japan; 2 12927 Gastroenterology, St Marianna University School of Medicine, Kawasaki, Japan; 3 13035 Department of Medical Oncology, Sapporo Medical University, Sapporo, Japan

**Keywords:** Endoscopic ultrasonography, Pancreas, Tissue diagnosis

## Abstract

**Background and study aims:**

Matched therapy based on comprehensive genomic profiling is a potential treatment option for patients with inoperable pancreatic cancer; however, the optimal method for obtaining tissue samples suitable for comprehensive genomic profiling using endoscopic ultrasound-guided tissue acquisition remains unclear. This study aimed to determine the optimal endoscopic ultrasound-guided tissue acquisition method to obtain samples for comprehensive genomic profiling.

**Patients and methods:**

This retrospective study included 86 consecutive patients with pancreatic cancer who underwent comprehensive genomic profiling using FoundationOne CDx (Foundation Medicine Inc.) and endoscopic ultrasound-guided tissue acquisition between June 2019 and January 2023. Macroscopic visible core length was measured using on-site macroscopic evaluation in all patients. Foundation Medicine Inc. reported analysis results categorized as passed (successful FoundationOne CDx), qualified, or failed. We investigated factors predicting successful FoundationOne CDx treatment.

**Results:**

Needles sized 22, 20, and 19 gauge were used in 63, one, and 23 patients, respectively. The stylet slow-pull and suction techniques were performed in 43 and 41 patients, respectively. Median total macroscopic visible core length in the formalin-fixed paraffin-embedded blocks subjected to FoundationOne CDx was 41 mm. The success rate for FoundationOne CDx was 66%. Multiple linear regression analysis revealed that macroscopic visible core length independently affected successful FoundationOne CDx ( *P* = 0.0019).

**Conclusions:**

In tissue specimens obtained using endoscopic ultrasound-guided tissue acquisition, macroscopic visible core length can be associated with an appropriate sample for FoundationOne CDx.

## Introduction

Incidence of pancreatic cancer has been increasing annually 1 2 . Pancreatic cancer is considered unresectable in 85% to 90% of cases at diagnosis and has a poor prognosis 3 . Recently, precision medicine has been implemented for pancreatic cancer and has been shown to improve prognosis of unresectable pancreatic cancer 4 . Comprehensive genomic profiling (CGP) of pancreatic cancer utilizes samples obtained through surgery, biopsy, endoscopic ultrasound-guided tissue acquisition (EUS-TA), and blood tests 5 6 . In Japan, CGP has been approved for coverage in patients with solid tumors that are refractory to standard chemotherapy.

EUS-TA is essential for diagnosing pancreatic cancer because of its high diagnostic accuracy 7 8 9 . Recent studies have demonstrated the utility of samples obtained using EUS-TA for CGP. However, the success rate for CGP is insufficient when obtained through EUS-TA (68%) compared with surgical specimens (95%) 10 .

Tumor tissue quantity and tumor nuclei ratio in submitted specimens are crucial for CGP 11 . Therefore, when performing CGP using samples obtained through EUS-TA, obtaining sufficient tumor tissue is important. Needle designs with innovative shapes, such as the Franseen needle, are more effective than conventional needles for tumor tissue diagnosis 11 12 . Recently, Takagi et al. 13 reported that the Franseen needle can ensure sufficient tissue acquisition compared with conventional needles for microsatellite instability evaluation in patients with pancreatic cancer. However, the issue of improving the success rate of CGP remains unresolved. This includes optimizing EUS-TA for CGP, such as needle size, aspiration technique, usefulness of macroscopic on-site evaluation (MOSE), and slide preparation method.

Measurement of macroscopic visible core (MVC) length during MOSE is useful in reducing the number of needle passes required for diagnosis, predicting the correct diagnosis, and increasing tumor tissue quantity 14 15 16 17 18 . However, whether MVC length is useful for predicting adequate tissue for CGP has not been well investigated.

The representative CGP used for this study was FoundationOne CDx (F1CDx) (Foundation Medicine, Inc., Cambridge, Massachusetts, United States). We previously investigated the optimal number of needle passes for F1CDx by EUS-TA without MOSE and found that two and four passes were optimal when using a 19 or 22G needle, respectively 19 . However, a limitation of the previous study was that assessment was only based on adequacy of pathological reports evaluated by a pathologist and not on results of F1CDx. Therefore, our previous results needed further confirmation via F1CDx. The current study aimed to elucidate the success rate of F1CDx for pancreatic cancer using samples obtained through EUS-TA with MOSE and investigate factors affecting success of F1CDx.

## Patients and methods

### Patients

This retrospective study was conducted at a tertiary referral cancer center, where approximately 300 to 400 EUS-TAs are performed annually. We reviewed consecutive patients with pancreatic cancer who were scheduled for F1CDx for the first time using tissues obtained via EUS-TA between June 2019 and January 2023. Patients who had undergone EUS-TA at other hospitals were excluded. This study was approved by our institutional review board (J2023–218–2023–1-3), and all patients provided informed consent for EUS-TA.

### EUS-TA procedure

Although most EUS-TA procedures are conducted when pancreatic masses need to be diagnosed pathologically, a few are performed for sample collection for F1CDx after chemotherapy induction. EUS-TA was performed under conscious sedation using a convex-array echoendoscope (GF-UCT260; Olympus Medical Systems Corp., Tokyo, Japan). All procedures were performed or supervised by an expert endoscopist who had performed more than 1000 EUS-TA procedures. In practice, EUS-TA procedures were performed by an expert or by multiple advanced endoscopy fellows under expert supervision. The mass was initially defined endosonographically, and the area was scanned using color Doppler to detect interposed vessels in the lesion. The type and size of the needle were selected at the endosonographer discretion: 19, 20, and 22G needles; Acquire (Boston Scientific Corporation, Natick, Massachusetts, United States); SonoTip TopGain (Medi-Globe GmbH; Rosenheim, Germany); SharkCore (Medtronic Corporation; Minneapolis, Minnesota, United States); and EchoTip ProCore HD (Wilson Cook Medical Inc.; Winston-Salem, NC, United States). A 10-mL suction or slow-pull technique was applied at endosonographer discretion. Under negative pressure suction, the needle was moved back and forth through the lesion approximately 10 to 20 times using the fanning method. At least two punctures were performed on each patient. Additional passes were performed if the obtained sample was deemed insufficient by MOSE. In our center, we did not perform rapid on-site evaluation.

### MOSE and slide preparation

**Fig. 1:**
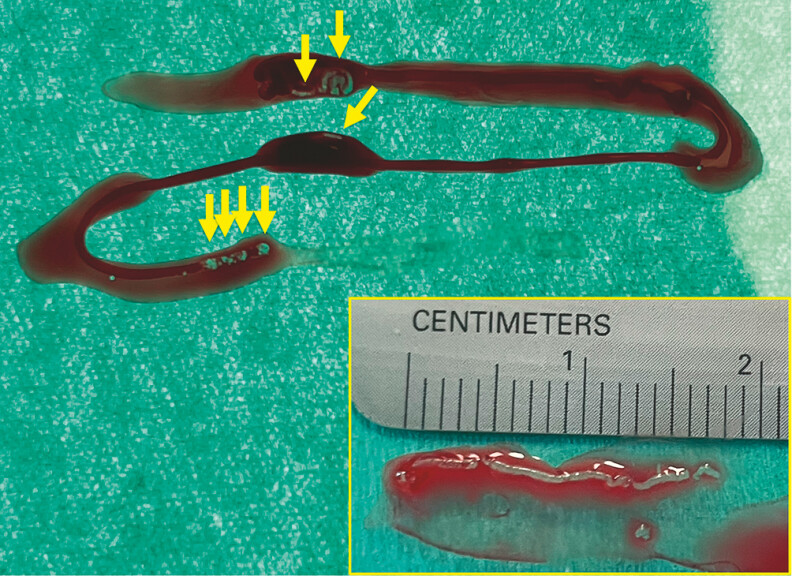
Macrosopic visible cores in the specimen (arrows) and measurement of macroscopic visible core length after aligning all scattered visible core fragments during macroscopic on-site evaluation.

Experienced endosonographers conducted standardized MOSE as described previously 17 19 . After removing the needle, the acquired material was placed in a Petri dish using a stylet. The MVC, defined as a measurable whitish sample as in our previous studies, was then trimmed, gathered, and aligned using a point-tip tweezer and a 23G injection needle 17 19 . The entire length of the MVC specimen was measured with a ruler and recorded per needle pass ( Fig. 1 ). The initial puncture sample was immersed in normal saline and sent to the Pathology Department. A portion of the first-pass MVC was placed on a glass slide and spread on another slide using the squash technique. Two smeared glass slides were stained with hematoxylin and eosin (HE) and Papanicolaou stains for cytological evaluation. The remaining samples from the first pass and the complete samples from the subsequent passes were fixed in 10% neutral-buffered formalin, paraffin-embedded, and sent for histological assessment. Two different slide preparations were performed during the study period, as described previously 19 . The reason for this modification was to increase the amount of samples in one formalin-fixed paraffin-embedded (FFPE) block for a successful F1CDx. Specifically, samples obtained from each needle pass between November 2019 and April 2020 were fixed and embedded separately (separate embedding). Consequently, a single FFPE block contained the sample obtained from a single needle pass, regardless of the number of passes. After May 2020, a single block combining all specimens (combined embedding) was prepared to increase tissue quantity per FFPE block, except for a small piece of first-pass MVC. HE-stained slides were prepared for histological diagnosis from each FFPE block. When submitting F1CDx, a pathologist selected the FFPE block containing the largest amount of tumor tissue. More than 10 unstained slides from this FFPE block were then submitted according to the instructions from Foundation Medicine.

### Definition of a successful F1CDx test

Foundation Medicine Inc. reported results of the F1CDx test categorized as passed, qualified, or failed. Qualified indicated that sensitivity for detecting genomic alterations and signatures may be reduced and that tumor mutation burden may be underreported. Passed was defined as a successful F1CDx test, and passed and qualified were defined as analyzable F1CDx test. Specimens deemed insufficient for F1CDx analysis by pathologists at our center were defined as unsuitable slides.

### Statistical analysis

The main outcome measure was the success rate of the F1CDx test, and factors that predicted a successful F1CDx test were investigated. Regarding methods used during EUS-TA, we focused on needle size, aspiration technique, MVC length, and slide preparation method. MVC length was defined as the sum of all MVC fragments contained within a single FFPE block submitted for F1CDx analysis. Categorical variables were compared using Fisher’s exact test. Continuous variables were compared using the Mann-Whitney U-test. In addition, multiple linear regression analysis of factors affecting successful F1CDx tests was performed. Because only a few studies have investigated factors influencing sample adequacy for CGP using EUS-TA, we selected factors for successful CGP based on previous studies examining factors associated with specimen size and quality of EUS-TA or our speculation 9 20 21 . Furthermore, receiver operating characteristic (ROC) curve analysis was used to estimate an optimal cut-off value of MVC length for a successful F1CDx, which might become a potential indicator for terminating tissue sampling for F1CDx. Furthermore, ROC curve analysis was used to estimate an optimal cut-off value of MVC length for a successful F1CDx, which might become a potential indicator for terminating tissue sampling for F1CDx. Statistical significance was set at *P* < 0.05 for all tests. Statistical analyses were performed using EZR version 4.0.2 (Saitama Medical Center, Jichi Medical University, Saitama, Japan), a graphical user interface for R (R Foundation for Statistical Computing, Vienna, Austria).

## Results

**Fig. 2:**
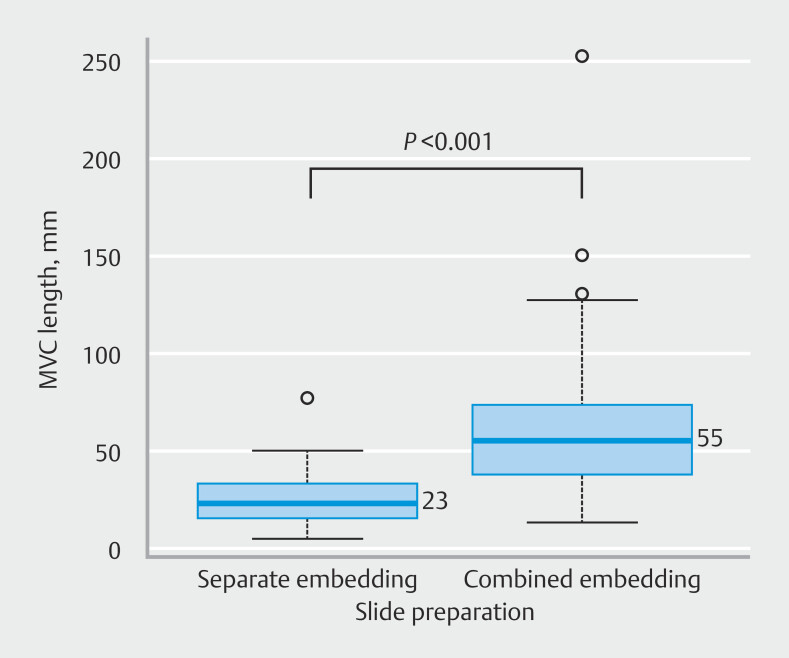
Relationship between macroscopic visible core length and slide preparation. MVC, macroscopic visible core.

**Fig. 3:**
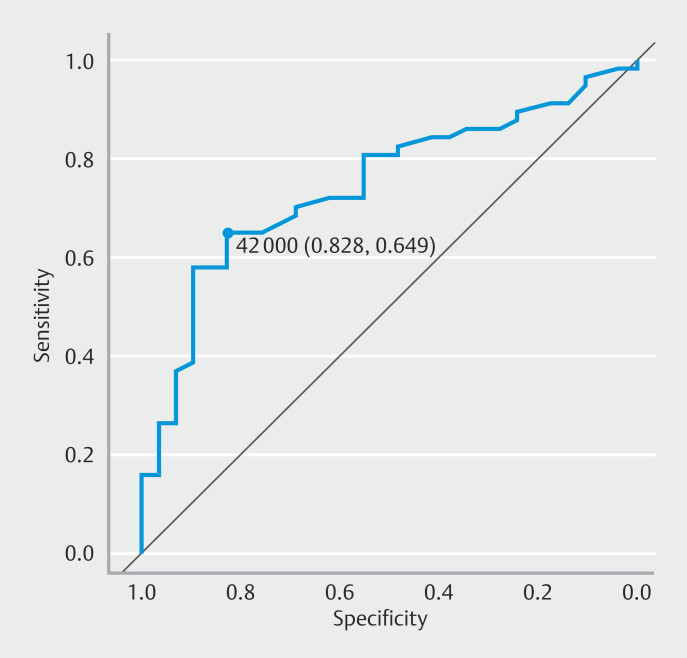
Receiver operating characteristic curve of the ideal macroscopic visible core length for successful FoundationOne CDx.

A total of 762 consecutive patients underwent EUS-TA for solid abdominal masses between June 2019 and January 2023. The first F1CDx test was scheduled for 86 patients with pancreatic cancer. Among them, one patient who underwent EUS-TA at another hospital was excluded. Baseline characteristics of study patients and EUS-TA are summarized in Table 1 . Median lesion size was 30 mm. Chemotherapy was administered to 15 patients before EUS-TA. Needle types selected for EUS-TA were 22G Acquire (Boston Scientific Corporation, Natick, Massachusetts, United States), 19G SonoTip TopGain, 22-G SonoTip TopGain (Medi-Globe GmbH; Rosenheim, Germany), 19G SharkCore (Medtronic Corporation; Minneapolis, Minnesota, United States), and 20G EchoTip ProCore HD (Wilson Cook Medical Inc.; Winston-Salem, NC, United States) in 44, 22, 18, one, and one patient, respectively. Stylet slow-pull, 10-mL suction, and other techniques were performed in 42, 41, and three patients, respectively. MVC was confirmed in all the samples. Median total MVC length in the FFPE blocks subjected to F1CDx was 41 mm. Separate and combined embedding were performed on specimens from 27 and 59 patients, respectively. Median total MVC length was significantly longer with combined embedding than with separate embedding (55 vs. 23 mm, *P* < 0.001) ( Fig. 2 ). Results for the F1CDx are summarized in Table 2 . Three slides were deemed unsuitable by pathologists at our center and could not be submitted to F1CDx owing to low tumor tissue quantity. Successful and analyzable F1CDx rates were 66% and 80%, respectively. Factors associated with successful F1CDx were investigated using multiple linear regression analyses ( Table 3 ), which revealed that only MVC length was a significant predictor of success of F1CDx ( *P* = 0.0019). From the ROC curve analysis, the cut-off value of 42 mm (area under the curve, 0.75; 95% confidence interval, 0.64–0.85) was identified as the ideal MVC length for successful F1CDx ( Fig. 3 ).

**Table:** **Table 1** Baseline characteristics of patients and EUS-TA.

		N = 86
Patient characteristics		
Median age, years (IQR)		66.5 (60.25–71.00)
Male:female		46:40
Prior chemotherapy, n		15 (17%)
Median lesion size, mm (IQR)		30.0 (23.25–33.00)
EUS-TA characteristics		
Needle size 22G/20G/19G		62 / 1 / 23
Median number of passes, n (IQR)		2 (2–3)
Aspiration technique, n	Stylet slow-pull	42 (49%)
	10-mL suction	41 (48%)
	Others	3 (3%)
Slide preparation, n	Separate embedding	27 (31%)
	Combined embedding	59 (69%)
Median MVC length, mm (IQR)		41.0 (30.00–65.25)
EUS-TA, endoscopic ultrasound-guided tissue acquisition; IQR, interquartile range; MVC, macroscopic visible core.		

**Table:** **Table 2** Results of F1CDx.

	N = 86
Successful F1CDx, n	57/86 (66%)
Categorization from the reports, n	
Passed	57 (66%)
Qualified	18 (21%)
Failed	8 (9%)
Unsuitable slide, n	3 (3%)
F1CDx, FoundationOne CDx.	

**Table:** **Table 3** Multiple linear regression analyses for variables affecting successful F1CDx.

	B	SE	β	P value
Chemotherapy before EUS-TA	0.21	0.15	0.075	0.16
Lesion size	-0.0030	0.0055	–0.028	0.59
22G needle	0.18	0.12	0.080	0.14
Slow-pull	0.15	0.13	0.078	0.24
Number of passes	–0.000030	0.13	–0.000021	1.00
Approach type: Transduodenal	–0.044	0.11	–0.022	0.68
Combined embedding	0.18	0.15	0.085	0.24
MVC length	0.003	0.0015	0.14	0.017
B, partial regression coefficient; SE, standard error; β, standardized partial regression coefficient; F1CDx, FoundationOne CDx; MVC, macroscopic visible core.				

## Discussion

We conducted a retrospective study on the success rate of F1CDx using specimens obtained via EUS-TA in patients who underwent F1CDx. The success rate of F1CDx was 66%. An MVC length > 42 mm was an independent factor associated with successful F1CDx, with a rate of 88%.

Few studies have examined suitability of EUS-TA specimens for CGP. Ikeda et al. reported that adequacy rates of specimens submitted to the NCC Oncopanel were 39% using a 22G needle and 56% using a 19G needle 22 . Similarly, Okuno et al. conducted a study on specimens subjected to F1CDx and reported adequacy rates of 56% with a 22G needle and 73% with a 19G needle 23 . Both studies concluded that when using EUS-TA specimens for CGP, the 19G needle was more suitable for collecting adequate specimens. However, these studies evaluated adequacy of specimens pathologically before CGP submission, and our study is the first to investigate the success rate of analysis based on actual F1CDx submissions using EUS-TA specimens. In addition, while rapid on-site evaluation was performed in two previous studies, our study conducted MOSE in all cases and measured MVC length, which is a notable difference and strength of our study 22 23 .

Unlike previous studies, the difference in needle size was not a factor associated with success of the analysis in our study, with longer MVC length being the only significant factor for successful F1CDx analysis. Because a previous study revealed that 19G needles can procure larger samples compared with 22G needles, it seems more plausible that a 19G would be better suited for F1CDx 24 . In our clinical practice, to successfully perform F1CDx, there is a possibility that efforts were made to increase the sample amount when using a 22G needle, which might have reduced the gap between the two needles. Furthermore, many punctures in this study were performed using a 22G needle, and further investigation is needed to compare 22 and 19G needles when submitting F1CDx 22 23 .

Our institution has frequently reported the usefulness of MOSE and MVC length measurements in EUS-TA. In 2023, we investigated the relationship between MVC length and F1CDx adequacy as judged by pathologists, wherein we reported that an MVC length ≥ 41 mm with a 19G needle and ≥ 35 mm with a 22G needle could indicate F1CDx adequacy 19 . In this study, based on the analysis results of specimens submitted for F1CDx, an MVC length > 42 mm in collected specimens was found to be a significant factor contributing to successful F1CDx, supporting the results of our previous study 19 . Furthermore, this study also revealed that the total MVC length in specimens subjected to F1CDx was significantly longer with combined embedding than with separate embedding (55 vs. 23 mm, *P* < 0.001). This result is understandable because, in combined embedding, MVC length refers to the total length of MVC fragments from all needle passes, whereas in separate embedding, it represents the MVC length of a single FFPE block containing the largest amount of sample. In this regard, a combined method may be more suitable for F1CDx submission.

Recently, EUS-TA has become indispensable for diagnosing pancreatic cancer. Furthermore, gene therapy has been suggested to improve prognosis of unresectable pancreatic cancer, making CGP and gene therapy increasingly essential in pancreatic cancer management. Therefore, specimen collection during EUS-TA for unresectable pancreatic cancer diagnosis will likely require consideration of subsequent CGP in the future. MOSE is a simple technique that requires no special equipment and incurs minimal costs. In addition, measuring MVC length takes only a few minutes.

This study has some limitations. First, this was a single-center, non-randomized, retrospective study, which may have introduced selection bias in needle selection and the number of passes. Second, detailed specimen quality data, such as tumor density, tumor area, and DNA quantity, were not considered. Specimen quality reports sent by Foundation Medicine Inc. for F1CDx submission were based on their categorization of passed, qualified, or failed, without a detailed description of the criteria used. Third, we did not consider interobserver differences in MVC length measurements.

## Conclusions

In conclusion, MVC length can be associated with an appropriate sample for F1CDx when using tissue specimens obtained through EUS-TA. Combined embedding is recommended to obtain a longer MVC in a single FFPE block.
